# Evolution and development of the mammalian jaw joint: Making a novel structure

**DOI:** 10.1111/ede.12426

**Published:** 2022-12-11

**Authors:** Neal Anthwal, Abigail S. Tucker

**Affiliations:** ^1^ Centre for Craniofacial and Regenerative Biology, Faculty of Dentisry Oral and Craniofacial Sciences London UK

**Keywords:** dentary, mammal evolution, temporomandibular joint

## Abstract

A jaw joint between the squamosal and dentary is a defining feature of mammals and is referred to as the temporomandibular joint (TMJ) in humans. Driven by changes in dentition and jaw musculature, this new joint evolved early in the mammalian ancestral lineage and permitted the transference of the ancestral jaw joint into the middle ear. The fossil record demonstrates the steps in the cynodont lineage that led to the acquisition of the TMJ, including the expansion of the dentary bone, formation of the coronoid process, and initial contact between the dentary and squamosal. From a developmental perspective, the components of the TMJ form through tissue interactions of muscle and skeletal elements, as well as through interaction between the jaw and the cranial base, with the signals involved in these interactions being both biomechanical and biochemical. In this review, we discuss the development of the TMJ in an evolutionary context. We describe the evolution of the TMJ in the fossil record and the development of the TMJ in embryonic development. We address the formation of key elements of the TMJ and how knowledge from developmental biology can inform our understanding of TMJ evolution.

## Graphical abstract

This review describes the evolution of the novel mammalian novel jaw joint (temporomandibular joint [TMJ]) in the fossil record and the development of the TMJ in embryonic development. It addresses the formation of key elements of the TMJ and how knowledge from developmental biology can inform the understanding of TMJ evolution.

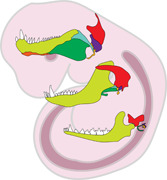

## 1 ARCHITECTURE OF THE TMJ

**Figure 1:**
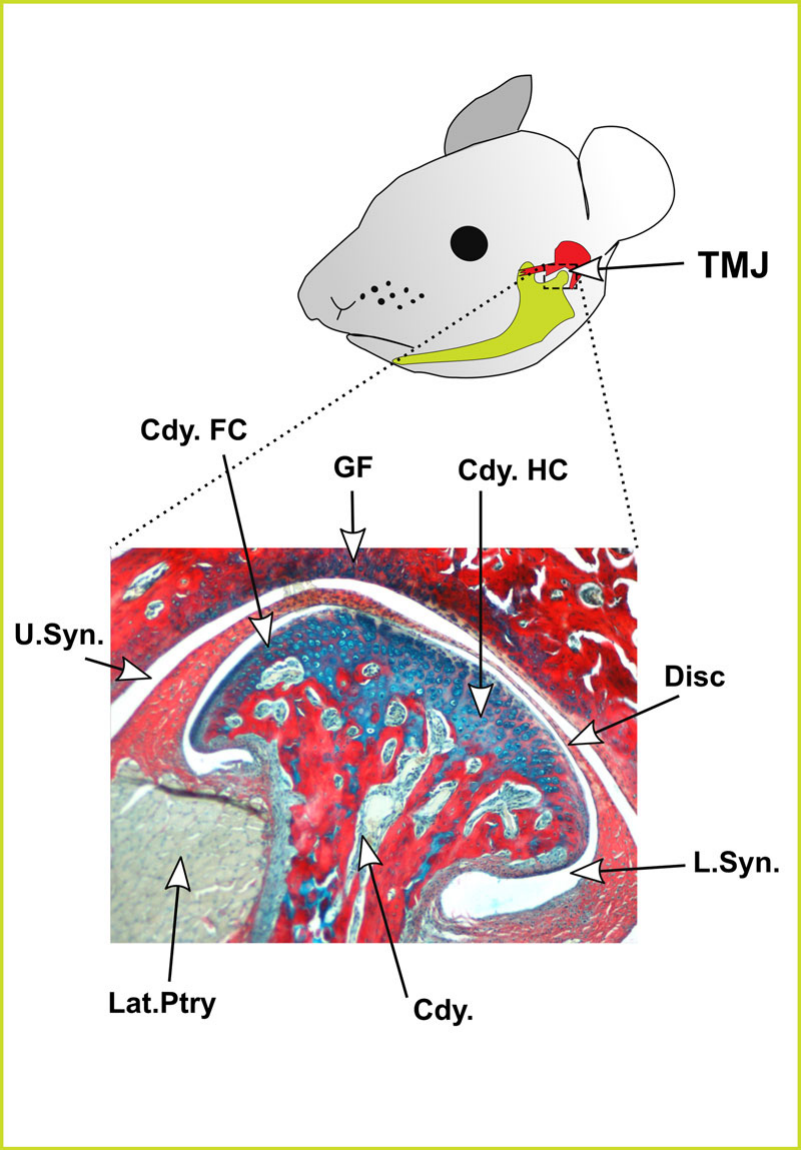
Anatomy of the mammalian jaw joint. Histological section through an adult mouse TMJ, stained with alizarin red (bone) and alcian blue (cartilage). Cdy, condylar process of mandible; Cdy.FC, fibrocartilage of condylar process; Cdy.HC, hyaline cartilage of condylar process; GF, glenoid fossa; Lat.Ptry lateral pterygoid muscle; L.Syn, lower synovial joint space; TMJ, temporomandibular joint; U.Syn, upper synovial joint space. [Color figure can be viewed at wileyonlinelibrary.com]

Mammals are defined by their novel squamosal‐dentary jaw articulation (Kemp, 2005), referred to as the temporomandibular joint (TMJ) in humans. This new jaw joint is formed by the coming together of two dermal (i.e., intramembranously ossifying) bones: the condylar process of the dentary bone in the mandible and the glenoid fossa of the squamosal bone in the skull. Although a temporal bone, formed of a fused squamosal and petrosal, is not found in all mammals, for clarity, we will refer to the mammalian jaw joint as the TMJ throughout this review. In nonmammalian vertebrates, the dentary is one of a number of bones that form the compound lower jaw and is often associated with the teeth. In contrast, the mammalian dentary forms the whole lower jaw, housing the teeth, forming the jaw joint (condylar process), and providing muscle attachment sites in the form of the coronoid and angular processes. The condylar process of the dentary is capped by the condylar cartilage, which forms the articulation site for the lower jaw (Figure 1). A fibrocartilage disc sits between the condylar cartilage and glenoid fossa, within a synovial capsule and acts as a cushion for the joint. The TMJ disc attaches to the superior head of the lateral pterygoid muscle anteriorly, and to ligaments posteriorly, including the discomallear ligament that runs through the capsule of the middle ear to link the malleus and TMJ disc. The mammalian TMJ replaced the primary jaw joint between the articular and quadrate, found in all nonmammalian vertebrates, with these bones adapting to new roles in the mammalian middle ear (Allin, 1975; Allin & Hopson, 1992).

The evolution of the mammalian TMJ is closely related to changes in the dentition and food processing that occurred early in synapsid (mammal lineage) evolution (Bhullar et al., 2019; DeMar & Barghusen, 1972; Gill et al., 2014; Grossnickle, 2017; Grossnickle et al., 2021; Sidor, 2003; Zhou et al., 2013). As well as having a novel jaw joint, mammals have teeth that differ from those of most other jawed vertebrates. These differences include a single replacement, rather than continual loss and replacement of teeth (diphydonty rather than polyphydonty), heterodonty (varied tooth shapes in different parts of the jaw) rather than homodonty (uniform tooth shapes throughout the jaw), and most importantly for the evolution of the TMJ, occlusion of the upper and lower dentition. Occlusion is facilitated by complimentary cusp patterns in the upper and lower dentition for shearing and crushing of food items (Crompton, 1981; Grossnickle et al., 2021; Jäger et al., 2020). The changes in the dentition would have put more strain on the primary jaw joint, leading to the added involvement of additional bones to buttress these effects and support the jaw during movement (Crompton & Hylander, 1986). Changes in dentition can, therefore, be thought of as the driving force for the creation of the new jaw joint. The dental and jaw changes together would have enabled greater processing of food in the oral cavity (i.e., increased mastication) and hence increased dietary efficiency for many foods (Bhullar et al., 2019; Grossnickel and Polly, 2013; Reilly et al., 2001). The new jaw joint and increasing redundancy of the primary jaw joint for mastication in turn permitted the increased and eventual total integration of the primary jaw joint into the hearing apparatus (Luo, 2011; Luo et al., 2016). A novel jaw, therefore, led to a novel ear.

Much of the recent literature has considered the evolution and development of the mammalian jaw and middle ear structures from the perspective of how the ancestral jaw articulation became integrated into the auditory apparatus. Little recent attention had been given to the formation of the novel joint itself, particularly from the evo‐devo perspective.

In this review, we will briefly describe the current knowledge of the formation of the TMJ (more thorough reviews on TMJ development are available elsewhere (Hinton, 2014; Hinton et al., 2015; W. E. Roberts & Stocum, 2018; Stocum & Roberts, 2018; Zhang et al., 2015)), and integrate this developmental data with the paleontological evidence for TMJ formation in the Mesozoic. We will discuss the relationship between the form and function of the TMJ and provide examples of the diversity of TMJ forms in extant mammals.

## 2 FORMATION OF AN ANATOMICAL NOVELTY

Several changes need to have occurred in order for the new anatomy of the mammalian TMJ to evolve from the basal amniote condition. The first likely change was the rearrangement of the muscles of mastication. Next, the dentary expanded to form both the condylar process and the attachment sites for the muscles of mastication. This was achieved by changes in the pattern of intramembranous ossification of the dentary, as well as by the important contribution of secondary cartilages (Anthwal & Tucker, 2012; Shibata et al., 2013; Shibata & Yokohama‐Tamaki, 2008; Vinkka, 1982). The squamosal then needed to form a fossa, to work with the condyle as the site for articulation in the cranial base, and finally, or possibly concurrently, the TMJ disc formed.

These changes cannot have occurred in isolation, and the development of each is partially dependent upon one or more of the other processes. For example, the development of the disc requires the development and function of the lateral pterygoid muscle (Anthwal & Tucker, 2020; Purcell et al., 2012), and the glenoid fossa forms through tissue interaction with the condylar cartilage (Li et al., 2021; Wang et al., 2011). These interdependent aspects will be discussed in more detail.

Novelties largely cannot emerge fully formed. Instead, they occur through co‐option, modification, or duplication of pre‐existing modules and developmental processes (Love, 2006; Moczek, 2008; Muller & Wagner, 1991; Shubin et al., 2009). The TMJ is an evolutionary novelty created from pre‐existing bones. In nonmammals, the dentary and squamosal dermal bones are present but form at a distance and never meet. The novelty is, therefore, that these bones have come together, interacted, and formed a functional new articulation. Similarly, the evolution of the TMJ has involved the possible co‐option of the tendon developmental program. The late appearance of the TMJ in vertebrate evolution is reflected by its late appearance during embryonic development. In vertebrate cranial skeletal development, the earliest developing structures are all cartilaginous. These are the cartilage precursors of the skull and face, known as the chondrocranium, and the cartilages of the pharyngeal arches. Many of these elements undergo endochondral ossification to form the bones of the face and cranial base, including the quadrate and articular of the primary jaw joint of nonmammals, and their homologues, the malleus incus of the mammal middle ear (Anthwal & Thompson, 2016; Tucker et al., 2004). The formation of the cartilaginous cranial endoskeleton is shortly followed by the initiation of the dermal skeleton of the skull vault and mandible, often referred to as the dermatocranium. Together the chondrocranium and dermatocranium represent the bauplan of the vertebrate skull. It is only following this common development that the secondary condylar cartilage forms in mammals, which is the first key step in TMJ initiation (Anthwal et al., 2008).

The late development of the TMJ is highlighted when studying mammals with extremely altricial births: the monotremes, and marsupials. In both of these mammalian groups, the young are born (marsupials) or hatch (monotremes) before the full ossification of the dentary, and before initiation of the condylar cartilage and so no TMJ is present. As such, both monotremes (Anthwal et al., 2020), and marsupials (Anthwal et al., 2017; Urban et al., 2017) use a mandibular middle ear as their craniomandibular articulation during early life. Once the TMJ has formed postnatally, the middle ear then separates from the mandible through a process of degradation of Meckel's cartilage (Anthwal et al., 2017; Urban et al., 2017). Monotremes appear to have a period of juvenile development where both primary and secondary (mammalian) cranial mandibular articulations are present (Anthwal et al., 2020; Ramírez‐Chaves et al., 2016; Watson, 1916; Zeller, 1993), an anatomical configuration similar to the form seen in mammalian ancestors, such as *Morganucodon* (Luo, 2011).

## 3 CHARTING TMJ EVOLUTION IN THE FOSSIL RECORD

**Figure 2:**
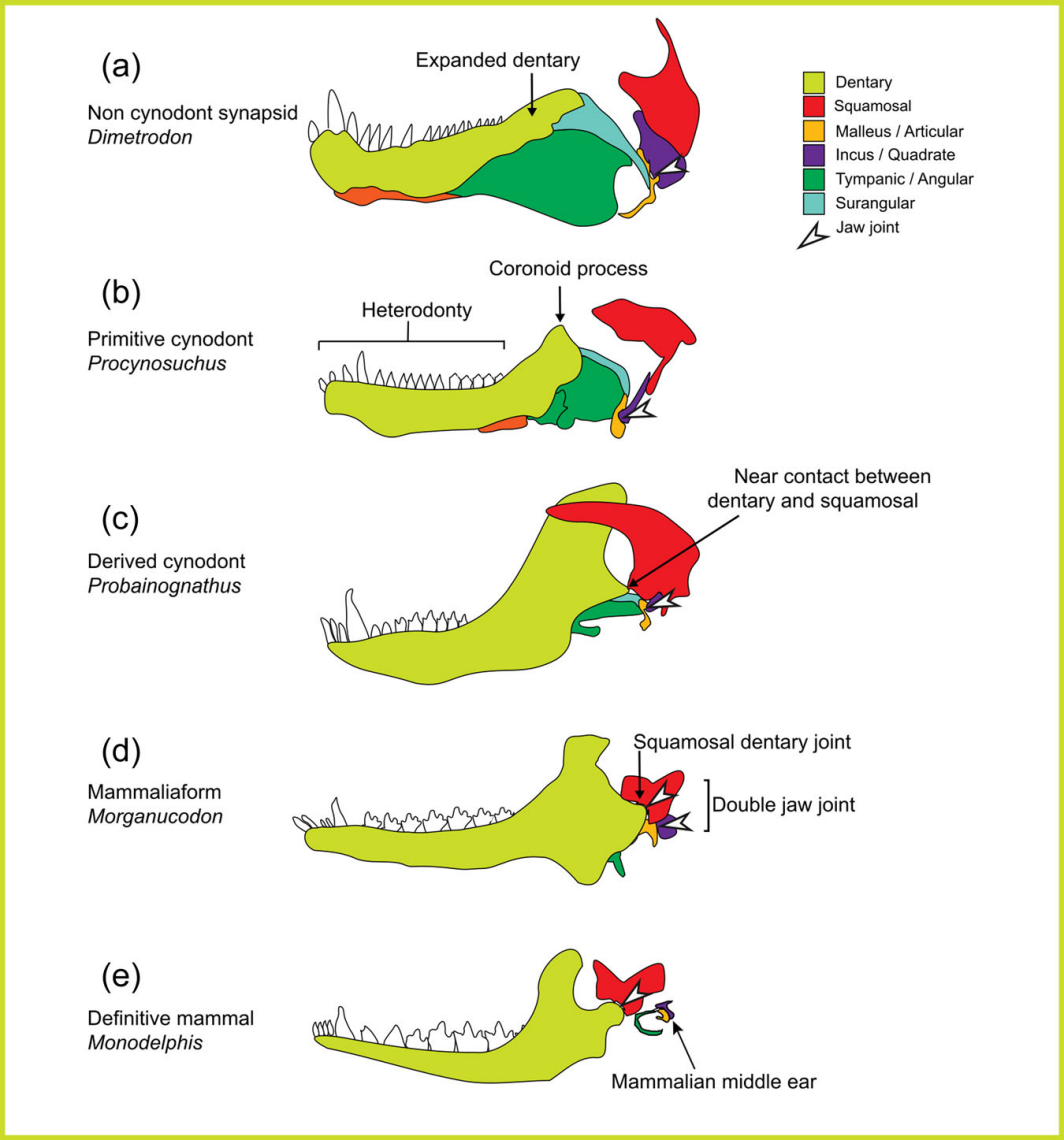
Key steps in TMJ evolution in the fossil record. Jaw joints in representatives of progressively more mammalian synapsids. Illustrations modified from the following sources: (a) after (Sidor, 2003), (b) after (Kemp, 1979), (c) after (Allin & Hopson, 1992), and (d, e) after (Anthwal et al. 2020). TMJ, temporomandibular joint. [Color figure can be viewed at wileyonlinelibrary.com]

As mentioned, the evolution of the novel mammalian jaw joint has been linked to the acquisition of diphyodont tribosphenic molars and the innovation of mammalian chewing (Figure 2), though the details of the mechanics of chewing in stem therian mammals remain debated (Bhullar et al., 2019, 2020; Grossnickle, 2017, 2020). Early noncynodont synapsids (Figure 2a), began to exhibit expanded dentaries and a progressive shrinking of the postdentary bones (Allin, 1975; Allin & Hopson, 1992; Kemp, 2005). This early dentary expansion included the development of a large and conspicuous coronoid process (Figure 2b). During development, the size of the coronoid is shaped by muscle action (Anthwal et al., 2015), indicating that the expansion during evolution was due to changes occurring in the masticatory muscles (Barghusen, 1968; Barghusen & Hopson, 1970; Crompton, 1972; Kemp, 2005). The expansion of the dentary and the creation of novel and larger muscle attachment sites (in the form of the angular and coronoid processes), before the formation of the squamosal–dentary connection, highlights that changes to the musculature predated changes to the joint. In keeping with this, it has been suggested that the forces developed by these anatomical changes resulted in the initiation of the squamosal dentary articulation, to act as a brace between the expanded dentary and the skull (Barghusen & Hopson, 1970). The earliest articulations between the dentary and squamosal have been found in cynodonts (Figure 2c). In brasilodontids and trieledontids, the dentary possessed a lateral ridge that contacted the squamosal but without forming a well‐developed articulation (Luo, 2011). Recently, a late Triassic brasilodontid (*Brasilodon quadrangularis*) has been identified as having a diphyodont mammalian dentition (Cabreira et al., 2022), confirming that acquisition of the mammalian dentition predates the jaw joint. Crucially, in brasilodontids and trieledontids, a dentary–squamosal interaction appears independently in separate lineages, implying it evolved multiple times (Barghusen & Hopson, 1970). This highlights the advantages offered by strengthening the connection between the upper and lower jaw in these early cynodonts. Other novel articulations have been observed in the fossil record but not in the lineage leading to mammals. The derived cynodont *Probainognathus*, for example, had the surangular bone interacting with the squamosal as well as near contact between the squamosal and dentary (Crompton, 1972). A clear double jaw joint (primary and dentary–squamosal) is evident in the Jurassic basal mammaliform *Morganucodon* (Figure 2d), along with the formation of a dentary condyle (Luo, 2011). The establishment of the novel cranial articulation freed up the primary articulation from its role in the jaw, allowing its separation from the dentary, as observed in the early Cretaceous *Liaoconodon*. Through the Cretaceous, early mammals showed increasing dominance of the TMJ, and increasing integration of the primary joint into the ear, ultimately leading to the definitive mammalian middle ear, as observed in modern mammals (Luo, 2011) (Figure 2e).

## 4 ROLE OF CRANIOFACIAL MUSCLE IN TMJ EVOLUTION AND DEVELOPMENT

Among the earliest adaptations of the stem‐mammal mandible was an alteration in the arrangement of the jaw closure musculature. The ancestral form of jaw closure muscles consisted of two subdivisions of the adductor muscle, m. adductor mandibulae externus and m. adductor mandibulae internus, while mammals have three, the masseter, the temporalis, and the lateral and medial pterygoids. This arrangement evolved early during mammalian evolution and is observed in basal cynodont mammal‐like reptiles, such as *Thrinaxodon* from the early Triassic, which has no secondary jaw joint (Lautenschlager et al., 2017). This new arrangement of jaw muscles in mammals and their ancestors allowed for a wider range of movement, including around the pivot point of the jaw, front to back, as well as side to side. In contrast, the jaw of nonmammals is more like a simple lever hinge (Reilly et al., 2001). The extra movements in mammals are enabled by a muscular sling around the dentary formed by the masseter and medial pterygoid muscles. The masseter muscle attaches to the outside of the mandible at the angular, creating a sling‐like anatomy not found in nonmammals, nor in the common amniote ancestor. Therefore, the migration of muscles from the internal jaw closure muscles to form the outer masseter was an essential early step in mammalian jaw joint evolution. This migration is well described in the fossil record (Crompton & Parker, 1978; Crompton, 1972).

Developmentally, the muscles of mastication responsible for opening and closing the jaw form from the cardiopharyngeal mesoderm and share their origin with cardiac muscles (Grenier et al., 2009; Ziermann et al., 2018). Both mammalian and nonmammalian muscles of mastication form from this developmental field, and their initial development depends on similar mechanisms (Michailovici et al., 2015). Later in development, the mammalian muscles separate into defined muscle groups with novel insertion sites, rather than the single adductor mandibulae proposed in the ancestral form. The mechanism of muscle migration is unclear. There are broadly two proposed models for the control of how muscle find their association with the skeleton, “nearest neighbor,” or “find and seek” (Diogo, Smith, et al., 2015; Diogo, Walsh, et al.,  2015). The muscles of the limb follow the “nearest neighbor” pattern, while most branchiomeric muscle exhibit “find and seek” activity, where signaling between the neural crest‐derived skeletal tissue and the mesodermal muscle tissue directs the muscle (Ziermann et al., 2018). Since the migrated mandibular site of muscle attachment is essential for the acquisition of the mammalian jaw joint, changes in the signals guiding the muscles must have also occurred. The precise control of muscle precursors to specific attachment sites is still unknown (although the control of attachment site development is an area of increasing research (Blitz et al., 2013; Eyal et al., 2015; Kult et al., 2021; Schweitzer et al., 2001; Sugimoto et al., 2013). In birds, the use of quail/duck transplants has revealed that neural crest cells can pattern the accompanying musculature, providing species‐specific attachment sites by changing spatiotemporal domains of gene expression (Tokita & Schneider, 2009). Changes in muscle development are, therefore, tightly connected with changes in the surrounding neural crest. Interestingly, experiments in mice have shown that loss of an ossified dentary does not prevent the muscles from migrating. In E18.5 mouse knockouts for the master regulator of osteogenesis *Runx2*, dermal bones such as the dentary do not form. However, the masseter, temporalis, and pterygoid muscles separate and find attachment sites in the condensed mesenchyme of the mandible (Shibata et al., 2004). Interestingly, in the absence of the dentary bone, the digastric and mylohyoid muscles of the base of the jaw, which would normally attach to the medial surface of the mandibular ramus, attach to ectopic extensions of Meckel's cartilage (Shibata et al., 2004). Meckel's cartilage is a rod of cartilage around which the bones of the mandible form, and which is largely transient in mammals, except the proximal‐most portion which forms the malleus and incus (Svandova et al., 2020). In mouse mutants lacking *Runx2* the muscles, therefore, attach to a structure that is transient in mammals, but persists in nonmammalian amniotes (Svandova et al., 2020).

Although the TMJ may have originally formed in response to mechanical stresses on the jaw, in extant mammals TMJ development is no longer constrained by the musculature or coronoid and angular processes. This is demonstrated by the formation of the TMJ in mouse mutants where either muscle or specific muscle attachment sites on the dentary are defective, but the TMJ still forms. These include *Pax9* mutant mice, which lack the coronoid process (Anthwal et al., 2015; Peters et al., 1998), *Tgf‐beta* mutants which fail to form the angular process (Anthwal et al., 2008), and *Tbx1* mutants with hypoplastic jaw musculature (Aggarwal et al., 2010; Anthwal et al., 2015). Similarly, the TMJ forms in mammals with reduced musculature, resulting in diminished dentary bones, such as anteaters, monotremes, and whales (El Adli & Deméré, 2015; Anthwal & Tucker, 2020; Cox & Jeffery, 2011; Naples, 1999). This demonstrates that the TMJ and musculature have a reduced developmental linkage than perhaps was the case in early mammals. The linkage between the musculature and the TMJ is not wholly severed, however, since the development of the TMJ disc is dependent on muscle tissue interactions (see later and (Anthwal & Tucker, 2020; Purcell et al., 2012).

## 5 SECONDARY CARTILAGE: CAPPING THE NEW JOINT

An important developmental change permitting the establishment of the mammal jaw joint was the formation of secondary cartilages. The condylar cartilage not only makes an important material contribution to the TMJ, acting as a growth center for the dentary but also signals to neighboring parts of the joint, including the glenoid fossa (Wang et al., 2011) and the disc, influencing their development (Bechtold et al., 2019; Purcell et al., 2009). While primary cartilages condense directly from undifferentiated mesenchyme before bone formation, secondary cartilages are so called since they form after, or secondarily to, the ossification of bone. They are found in mammals (Beresford, 1981; Hirouchi et al., 2017; Vinkka, 1982; Vinkka‐Puhakka & Thesleff, 1993), as well as dinosaurs (non‐avian (Bailleul et al., 2013) and avian, including extant birds (Bailleul et al., 2012; Solem et al., 2011)), and some teleost fish (Gillis et al., 2006). This divergent spread of secondary cartilage across clades, and their absence in all reptiles and amphibians, suggests that the presence of secondary cartilage is convergent in extant mammals, birds, and fish.

In birds, the articular secondary cartilages form directly from the periosteum and can be thought of as extensions of the bone, while the enthesis secondary cartilages form within the fibrous aponeurosis (Eames et al., 2003; Solem et al., 2011). In mammals, secondary cartilages appear to develop as independent condensations, close to the periosteum of the dermal bones to which they attach (Anthwal et al., 2008; Vinkka‐Puhakka & Thesleff, 1993), although counter‐evidence suggests that they are part of the periosteum (Shibata et al., 2013). In birds, the secondary cartilages form in response to a combination of mechanical stimulation and molecular signaling, mainly Tgf‐beta and Fgf (Solem et al., 2011; Woronowicz et al., 2018). The secondary cartilage which forms the condylar in mammals also requires Tgf‐beta signaling (Anthwal et al., 2008; Oka et al., 2007), but forms before the dentary and squamosal come into close articulation. In contrast to birds, mammalian secondary cartilages are able to develop in the absence of muscle or muscle activity and, therefore, do not require mechanical force to initiate (Anthwal et al., 2008, 2015; Rot‐Nikcevic et al., 2007).

On the other side of the joint from the condylar is the glenoid fossa, an inverted cup‐shaped articulation site on the squamosal, lined with fibrocartilage. Like the condylar, the cartilage of the glenoid fossa forms on an intramembranously ossifying bone. In mouse knock‐out studies, genetic dislocation of the TMJ during development results in a failure of the formation of the correct shape of the glenoid fossa (Wang et al., 2011). Fossa formation, therefore, appears dependent on tissue interactions with the rest of the joint, with the condylar helping to create its own socket, thereby allowing a precise fit during joint formation.

From the fossil record, it appears that the expanding dentary contacted the squamosal initially acting as a brace (Barghusen & Hopson, 1970). Later in evolution, secondary cartilage then capped these structures to create a more effective articulation surface (Kermack et al., 1973; Luo, 2011; Romer, 1969). In living mammals, the condylar cartilage not only creates articulation but also acts as an important growth center for the dentary (Baume, 1962; Frommer, 1964; Hinton, 2014; Hinton et al., 2016; Jing et al., 2015; Kantomaa & Hall, 1988; Shibukawa et al., 2007). This was likely the case in cynodonts with a fully formed TMJ. This is a unique aspect of mammalian secondary cartilage. When present, nonmammalian secondary cartilage does not contribute to the normal growth of bone, while the mammal condylar cartilage is a major site of mandibular growth (Anthwal & Tucker, 2012). It does this through endochondral ossification, and the chondrocytes of the condyle transdifferentiate into the osteocytes of the mandibular bone (Hinton et al., 2016; Jing et al., 2015; Ruscitto et al., 2020). Therefore, the formation of a novel secondary cartilage on the condylar process on the dentary was an essential step in TMJ evolution, providing both an articulation surface and a means for increasing mandible size, allowing further reshaping of the dentary.

## 6 THE TMJ DISC: A CUSHION TO SOFTEN THE BLOW

The TMJ disc is a unique component of the mammalian jaw joint: a fibrocartilage that is classically described as acting as a cushion between the mandible and skull. It is connected by ligaments to the otic capsule, and by a tendon to the superior head of the lateral pterygoid muscle. The disc develops from a condensation of mesenchyme cells overlying the cartilage of the condylar, which is also formed by fibrocartilage. Evidence in the mouse indicates that these cells initially are attached to the condylar cartilage, and later separate into the synovial cavity, with the upper cavity forming first followed by the lower (Purcell et al., 2009, 2012). Whether the upper or lower cavity forms first may be species‐specific as studies in human fetal tissues indicate that the lower cavity forms first, and the upper cavity forms secondarily (Mérida‐Velasco et al., 2009; 1999; Öğütcen‐Toller & Keskin, 2000). A similar sequence is observed in miniature pigs (Xiang et al., 2022).

Given the fibrocartilage nature of the disc and condyle, and their relative positions during development, the disc has been suggested to originate from the top layer of the condylar cartilage (Purcell et al., 2009). The disc would, therefore, be derived from secondary cartilage. Alternatively, it has been suggested that the disc has its origins as a sesamoid within a tendon caught between the elements of the evolving novel articulation, an idea first suggested by Gaupp (1911). This hypothesis remains controversial. Studies in multiple species have suggested connections between the developing lateral pterygoid muscle and malleus via the forming disc, while others find no such connection (Cheynet et al., 2003; Coleman, 1970; Harpman & Woollard, 1938; Pinto, 1962; Rodríguez‐Vázquez et al., 1998). Using human tissue, Ögütcen‐Toller and Juniper (1993) were able to identify a band of connecting mesenchyme between the developing lateral pterygoid and Meckel's cartilage (which forms the malleus) in 10‐week‐old human tissue. This band later is observed to pass through the medial aspect of the condylar en route to the malleus.

### 6.1 Developmental evidence for the tendon hypothesis

A tendon origin of parts of the TMJ is supported by the link with the key tendogenic transcription factor Scleraxis (Scx). Scx is expressed in developing tendons and ligaments, as well as in the bifated cells that form the bony enthesis, tuberosities, and sesamoids (Blitz et al., 2013; Eyal et al., 2015; Kult et al., 2021; Schweitzer et al., 2001; Sugimoto et al., 2013). Scx is expressed in both the condylar cartilage (Ma et al., 2021) and the disc (R. R. Roberts et al., 2019).

Further evidence comes from mouse knock‐out studies where the condylar cartilage is disrupted. Wang et al. (2011) showed that when the master regulator of cartilage, Sox9, is genetically removed from neural crest cells, the condylar cartilage failed to form. However, a disc‐like structure still formed connected to the lateral pterygoid muscle overlying the mandibular ramus at the condylar process where the cartilage was absent, suggesting that at least part of the disc is independent of cartilage in origin.

Under mechanical compressive and shear forces, tendons form sesamoids and enthesis eminences—hard tissues encapsulated within the tendon. These sesamoids form as both cartilage and bone. Fibrocartilaginous regions are mainly found in joints and in tendons that are subject to compression (Benjamin & Ralphs, 1998). Joint fibrocartilages include the meniscus of the knee, while fibrocartilages in tendons include those that pass around bony pulleys such as the flexor digitorum profundus tendon in quadrupeds, the suprapatellar tendon in rats, and tendon of extensor digitorum in the human hand. In addition, fibrocartilages form in the enthesis of long bones. Interestingly, it has been demonstrated that both the fibrocartilage TMJ disc and enthesis fibrocartilage cells in other parts of the body are derived from Hh‐responsive cells and that these cells are responsive to mechanical loading (Schwartz et al., 2015). The disc can mineralize due to age or after genetic manipulation in mouse mutants, for example, ectopic cartilage formed in the disc in *Proteoglycan4* mutant mice (Bechtold et al., 2016), in keeping with the disc having sesamoid‐like characteristics. An important aspect of the disc, in contrast to a sesamoid, is therefore retention of its fibrous identity.

Recent studies into the development of tendon–bone interfaces, or entheses, suggest that hybrid or bipotential cells are present that express both Scx tendon and Sox9 cartilage markers. Furthermore, the use of a tamoxifen‐inducible *ScxCre* transgenic mouse has indicated Scx lineage cells contribute to the cartilage of the condylar process (Ma et al., 2021). Following early postnatal Cre induction, this study demonstrated the potential of Scx‐expressing cells in the fibrocartilage layer of the condylar to contribute to the condylar cartilage, but interestingly these cells did not transdifferentiate into osteocytes during endochondral ossification while neighboring Scx negative condylar fibrocartilage cells did (Ma et al., 2021). The common Scx lineage of both disc and condyle suggests that both elements may have their origin in the connective tissue of the region, rather than the primary skeleton. In this way, they may be similar to entheses, such as the greater trochanter in the femur, and sesamoids, such as the patella, which are both derived from Scx (tendon) and Sox9 (cartilage) cellular lineages (Blitz et al., 2013; Eyal et al., 2015; Sugimoto et al., 2013).

### 6.2 Ecological adaptations

Mammals have occupied a range of dietary niches, and since the TMJ is largely unconstrained by other influences, adaptations in the different modules of TMJ reflect diet. These adaptations include loss of the disc in edentulous mammals, loss of the synovial cavity in some baleen whales, the mesolateraly aligned hinge‐like joint morphology in carnivorans, and the differential positioning of the joint in relation to the tooth row in carnivores versus herbivores. Each of the above adaptations has a developmental basis since they involve variations in anatomy. Due to the limitation of many model organisms, the joint angle and morphology examples above have not been investigated in a developmental context; however, the presence or absence of the TMJ disc has been.

Many species without teeth do not form a TMJ disc. These include some baleen whales, giant anteaters, and extant monotremes. In the case of monotremes, it was unclear if the absence of the TMJ disc was related to the absence of teeth, or whether the disc was a novelty of therian mammals (a clade that excludes monotremes). However, developmental studies using archival slides of platypus and echidna development have shown that monotremes initially form a disc in a similar manner to eutherians (Anthwal & Tucker, 2020). A disc anlage is observed as a thickening of mesenchymal cells on the surface of the condylar, but it later fails to separate into the synovial cavity. This anatomy is remarkably similar to the TMJ phenotype of *Mesp1Cre; Tbx1Flox* mouse mutants who have reduced musculature (Anthwal & Tucker, 2020). Similar phenotypes have been observed in a surgical study where the mouse mandible was sewn shut in utero (Habib et al., 2007). The TMJ disc, therefore, initiates in all mammals but its maturation is dependent on muscle activity. Mammals with reduced or absent teeth no longer require large muscles of mastication, which in turn has the knock‐on consequence of a poorly formed disc. Whether this change in the disc is due to a reduction in biomechanical force or signaling molecules coming from the muscles is unclear. Both physical contraction and signals from the muscle, such as Fgfs (Purcell et al., 2012), have been shown to shape disc formation, thus changes in the size of muscles can impact both force and signaling.

## 7 CONCLUSIONS

This review has used fossil, anatomical, and developmental data to provide an understanding of how and why the mammalian TMJ formed. Changes in the dentition are proposed to have led to changes in the musculature, impacting the shape of the dentary with the formation of new muscle attachment sites. Buttressing of the dentary against the upper jaw to support mastication led to the formation of a new point of articulation, with the capping of the dentary with secondary cartilage and signaling between the upper and lower jaw leading to the creation of a robust TMJ joint. Potential trapping of a tendon resulted in the creation of the TMJ disc, allowing cushioning of jaw movements, while the secondary cartilage of the condylar additionally provided a new growth center for further expansion of the dentary. As a unique feature of mammals, the TMJ has resulted in the evolution of the mammalian middle ear, as well as increasing food processing efficiency and allowed for an expansion of the mammalian diet. Importantly, the presence of the new joint enabled mammals to exploit the increase in herbivore niches following the angiosperm radiation (Grossnickle & Polly, 2013). Changes to the mammalian jaw joint have, therefore, been important factors in the remarkable radiation of mammals into a wide range of ecological niches.

Despite advances in our understanding of TMJ evolution and development highlighted here, there are still a number of gaps in our knowledge. In particular, how mammalian jaw joint diversity is established is poorly understood. For example, the shape of the condylar head and glenoid fossa varies in mammals of differing diets. Carnivores have condylar heads that are wide in the medial‐lateral axis, and deep glenoid fossae resulting in hinge‐like joints, while herbivores have shallower fossae to better aid lateral movement during chewing. How these differences are established in mammals is largely unknown, however, interspecies grafts in birds have highlighted that the cranial neural crest provides species‐specific patterning information, controlling both jaw shape and size (Schneider, 2018; Schneider & Helms, 2003; Tucker & Lumsden, 2004). The cranial neural crest is, therefore, likely to be a central driver of this process also in mammals, providing patterning information and coordinating muscle and skeletal interactions. To answer such questions and others, the expansion of the organisms used in developmental biology will prove invaluable (see also Howenstine et al., 2021).

## Data Availability

No novel data was presented.

## References

[ede12426-bib-0001] El Adli, J. J. , & Deméré, T. A. (2015). On the anatomy of the temporomandibular joint and the muscles that act upon it: Observations on the gray whale, *Eschrichtius robustus* . The Anatomical Record, 298, 680–690. 25737135 10.1002/ar.23109

[ede12426-bib-0002] Aggarwal, V. S. , Carpenter, C. , Freyer, L. , Liao, J. , Petti, M. , & Morrow, B. E. (2010). Mesodermal Tbx1 is required for patterning the proximal mandible in mice. Developmental Biology, 344, 669–681. 20501333 10.1016/j.ydbio.2010.05.496 PMC2917794

[ede12426-bib-0003] Allin, E. F. (1975). Evolution of the mammalian middle ear. Journal of Morphology, 147(4), 403–437. 1202224 10.1002/jmor.1051470404

[ede12426-bib-0004] Allin, E. F. , & Hopson, J. A. (1992). Evolution of the auditory system in Synapsida (“mammal‐like reptiles” and primitive mammals) as seen in the fossil record. In eds. D. B. Webster , R. R. Fay & A. N. Popper , The evolutionary biology of hearing (pp. 587–614). Springer‐Verlag.

[ede12426-bib-0005] Anthwal, N. , Chai, Y. , & Tucker, A. S. (2008). The role of transforming growth factor‐β signalling in the patterning of the proximal processes of the murine dentary. Developmental Dynamics, 237, 1604–1613. 18498113 10.1002/dvdy.21567

[ede12426-bib-0006] Anthwal, N. , Fenelon, J. C. , Johnston, S. D. , Renfree, M. B. , & Tucker, A. S. (2020). Transient role of the middle ear as a lower jaw support across mammals. eLife, 9, 1–36. 10.7554/eLife.57860 PMC7363448 32600529

[ede12426-bib-0007] Anthwal, N. , Peters, H. , & Tucker, A. S. (2015). Species‐specific modifications of mandible shape reveal independent mechanisms for growth and initiation of the coronoid. EvoDevo, 6, 35. 26568815 10.1186/s13227-015-0030-6 PMC4644282

[ede12426-bib-0008] Anthwal, N. , & Thompson, H. (2016). The development of the mammalian outer and middle ear. Journal of Anatomy, 228, 217–232. 26227955 10.1111/joa.12344 PMC4718165

[ede12426-bib-0009] Anthwal, N. , & Tucker, A. S. (2012). Molecular biology of the mammalian dentary: Insights into how complex skeletal elements can be shaped during development and evolution. In R. J. Asher , & J. Müller (Eds.), From clone to bone: The synergy of morphological and molecular tools in palaeobiology (pp. 207–229). Cambridge University Press.

[ede12426-bib-0010] Anthwal, N. , & Tucker, A. S. (2020). The TMJ disc is a common ancestral feature in all mammals, as evidenced by the presence of a rudimentary disc during monotreme development. Frontiers in Cell and Developmental Biology, 8, 356. 32509783 10.3389/fcell.2020.00356 PMC7248220

[ede12426-bib-0011] Anthwal, N. , Urban, D. J. , Luo, Z.‐X. , Sears, K. E. , & Tucker, A. S. (2017). Meckel's cartilage breakdown offers clues to mammalian middle ear evolution. Nature Ecology & Evolution, 1, 93. 28459103 10.1038/s41559-017-0093 PMC5405799

[ede12426-bib-0012] Bailleul, A. M. , Hall, B. K. , & Horner, J. R. (2012). First evidence of dinosaurian secondary cartilage in the post‐hatching skull of *Hypacrosaurus stebingeri* (Dinosauria, Ornithischia). PLoS One, 7, e36112. 22558351 10.1371/journal.pone.0036112 PMC3340333

[ede12426-bib-0013] Bailleul, A. M. , Hall, B. K. , & Horner, J. R. (2013). Secondary cartilage revealed in a non‐avian dinosaur embryo. PLoS One, 8, e56937. 23418610 10.1371/journal.pone.0056937 PMC3572077

[ede12426-bib-0014] Barghusen, H. (1968). The lower jaws of cynodonts (Reptilia, Therapsida) and the evolutionary origin of mammal‐like adductor jaw musculature. Postilla, 116, 1–49.

[ede12426-bib-0015] Barghusen, H. R. , & Hopson, J. A. (1970). Dentary‐squamosal joint and the origin of mammals. Science, 168, 573–575. 5436589 10.1126/science.168.3931.573

[ede12426-bib-0016] Baume, L. J. (1962). Ontogenesis of the human temporomandibular joint. 1. Development of the condyles. Journal of Dental Research, 41(6), 1327–1339. 13966993 10.1177/00220345620410060901

[ede12426-bib-0017] Bechtold, T. E. , Kurio, N. , Nah, H.‐D. , Saunders, C. , Billings, P. C. , & Koyama, E. (2019). The roles of Indian Hedgehog signaling in TMJ formation. International Journal of Molecular Sciences, 20, 6300. 31847127 10.3390/ijms20246300 PMC6941023

[ede12426-bib-0018] Bechtold, T. E. , Saunders, C. , Mundy, C. , Um, H. , Decker, R. S. , Salhab, I. , Kurio, N. , Billings, P. C. , Pacifici, M. , Nah, H. D. , & Koyama, E. (2016). Excess BMP signaling in heterotopic cartilage forming in Prg4‐null TMJ discs. Journal of Dental Research, 95, 292–301. 26534931 10.1177/0022034515613508 PMC4766954

[ede12426-bib-0019] Benjamin, M. , & Ralphs, J. R. (1998). Fibrocartilage in tendons and ligaments‐‐an adaptation to compressive load. Journal of Anatomy, 193(4), 481–494. 10029181 10.1046/j.1469-7580.1998.19340481.x PMC1467873

[ede12426-bib-0020] Beresford, W. A. (1981). Chondroid bone, secondary cartilage, and metaplasia. Urban & Schwarzenberg.

[ede12426-bib-0021] Bhullar, B.‐A. S. , Manafzadeh, A. R. , Miyamae, J. A. , Hoffman, E. A. , Brainerd, E. L. , Musinsky, C. , & Crompton, A. W. (2019). Rolling of the jaw is essential for mammalian chewing and tribosphenic molar function. Nature, 566, 528–532. 30760927 10.1038/s41586-019-0940-x

[ede12426-bib-0022] Bhullar, B.‐A. S. , Manafzadeh, A. R. , Miyamae, J. A. , Hoffman, E. A. , Brainerd, E. L. , Musinsky, C. , & Crompton, A. W. (2020). Reply to: Jaw roll and jaw yaw in early mammals. Nature, 582, E9–E12. 32555494 10.1038/s41586-020-2364-z

[ede12426-bib-0023] Blitz, E. , Sharir, A. , Akiyama, H. , & Zelzer, E. (2013). Tendon‐bone attachment unit is formed modularly by a distinct pool of Scx‐ and Sox9‐positive progenitors. Development, 140, 2680–2690. 23720048 10.1242/dev.093906

[ede12426-bib-0024] Cabreira, S. F. , Schultz, C. L. , da Silva, L. R. , Lora, L. H. P. , Pakulski, C. , do Rêgo, R. C. B. , Soares, M. B. , Smith, M. M. , & Richter, M. (2022). Diphyodont tooth replacement of brasilodon—A late triassic eucynodont that challenges the time of origin of mammals. Journal of Anatomy, 241, 1424–1440. 36065514 10.1111/joa.13756 PMC9644961

[ede12426-bib-0025] Cheynet, F. , Guyot, L. , Richard, O. , Layoun, W. , & Gola, R. (2003). Discomallear and malleomandibular ligaments: Anatomical study and clinical applications. Surgical and Radiologic Anatomy, 25, 152–157. 12802513 10.1007/s00276-003-0097-y

[ede12426-bib-0026] Coleman, R. D. (1970). Temporomandibular joint: Relation of the retrodiskal zone to Meckel's cartilage and lateral pterygoid muscle. Journal of Dental Research, 49, 626–630. 5269119 10.1177/00220345700490032701

[ede12426-bib-0027] Cox, P. G. , & Jeffery, N. (2011). Reviewing the morphology of the jaw‐closing musculature in squirrels, rats, and Guinea pigs with contrast‐enhanced microCT. The Anatomical Record: Advances in Integrative Anatomy and Evolutionary Biology, 294, 915–928. 21538924 10.1002/ar.21381

[ede12426-bib-0028] Crompton, A. W. (1972). The evolution of the jaw articulation of cynodonts. In K. A. Joysey & T. S. Kemp (Eds.), Studies in Vertebrate Evolution, (pp. 231–251). Oliver & Boyd.

[ede12426-bib-0029] Crompton, A. W. (1981). The origin of mammalian occlusion. In H. G. Barrer (Ed.), Orthodontics: The state of the art (pp. 3–18). University of Pennsylvania Press.

[ede12426-bib-0030] Crompton, A. W. , & Hylander, W. L. (1986). Changes in mandibular function following the acquisition of a dentary‐squamosal joint. In N. Hotton , P. D. Maclean , J. J. Roth , & E. C. Roth (Eds.), The ecology and biology of mammal‐like reptiles (pp. 78–98). Smithsonian Institute Press.

[ede12426-bib-0031] Crompton, A. W. , & Parker, P. (1978). Evolution of the mammalian masticatory apparatus. American Scientist, 66, 192–201. 646211

[ede12426-bib-0032] DeMar, R. , & Barghusen, H. R. (1972). Mechanics and the evolution of the synapsid jaw. Evolution, 26(4), 622–637. 28563363 10.1111/j.1558-5646.1972.tb01969.x

[ede12426-bib-0033] Diogo, R. , Smith, C. M. , & Ziermann, J. M. (2015). Evolutionary developmental pathology and anthropology: A new field linking development, comparative anatomy, human evolution, morphological variations and defects, and medicine. Developmental Dynamics, 244, 1357–1374. 26293597 10.1002/dvdy.24336

[ede12426-bib-0034] Diogo, R. , Walsh, S. , Smith, C. , Ziermann, J. M. , & Abdala, V. (2015). Towards the resolution of a long‐standing evolutionary question: muscle identity and attachments are mainly related to topological position and not to primordium or homeotic identity of digits. Journal of Anatomy, 226, 523. 25851747 10.1111/joa.12301 PMC4450956

[ede12426-bib-0115] Eames, B. F. , De La Fuente, L. , & Helms, J. A. (2003). Molecular ontogeny of the skeleton. Birth Defects Research Part C: Embryo Today: Reviews, 69(2), 93–101. 12955855 10.1002/bdrc.10016

[ede12426-bib-0035] Eyal, S. , Blitz, E. , Shwartz, Y. , Akiyama, H. , Schweitzer, R. , & Zelzer, E. (2015). On the development of the patella. Development, 142, 1831–1839. 25926361 10.1242/dev.121970

[ede12426-bib-0036] Frommer, J. (1964). Prenatal development of the mandibular joint in mice. The Anatomical Record, 150(4), 449–461. 14248316 10.1002/ar.1091500414

[ede12426-bib-0037] Gaupp, E. (1911). Beiträge zur Kenntnis des Unterkiefers der Wirbeltiere. I. Der Processus anterior (Folii) des Hammers der Säuger und das Goniale der Nichtsäuger. Anatomischer Anzeiger, 39, 97–135.

[ede12426-bib-0038] Gill, P. G. , Purnell, M. A. , Crumpton, N. , Brown, K. R. , Gostling, N. J. , Stampanoni, M. , & Rayfield, E. J. (2014). Dietary specializations and diversity in feeding ecology of the earliest stem mammals. Nature, 512(7514), 303–305. 25143112 10.1038/nature13622

[ede12426-bib-0039] Gillis, J. A. , Witten, P. E. , & Hall, B. K. (2006). Chondroid bone and secondary cartilage contribute to apical dentary growth in juvenile Atlantic salmon. Journal of Fish Biology, 68, 1133–1143.

[ede12426-bib-0040] Grenier, J. , Teillet, M.‐A. , Grifone, R. , Kelly, R. G. , & Duprez, D. (2009). Relationship between neural crest cells and cranial mesoderm during head muscle development. PLoS One, 4, e4381. 19198652 10.1371/journal.pone.0004381 PMC2634972

[ede12426-bib-0041] Grossnickle, D. M. (2017). The evolutionary origin of jaw yaw in mammals. Scientific Reports, 7, 45094. 28322334 10.1038/srep45094 PMC5359619

[ede12426-bib-0042] Grossnickle, D. M. (2020). Jaw roll and jaw yaw in early mammals. Nature, 582, E6–E8. 32555493 10.1038/s41586-020-2365-y

[ede12426-bib-0043] Grossnickle, D. M. , & Polly, P. D. (2013). Mammal disparity decreases during the Cretaceous angiosperm radiation. Proceedings of the Royal Society B: Biological Sciences, 280, 20132110. 10.1098/rspb.2013.2110 PMC3790494 24089340

[ede12426-bib-0044] Grossnickle, D. M. , Weaver, L. N. , Jäger, K. R. K. , & Schultz, J. A. (2021). The evolution of anteriorly directed molar occlusion in mammals. Zoological Journal of the Linnean Society, 194, 349–365 .

[ede12426-bib-0045] Habib, H. , Hatta, T. , Rahman, O. I. F. , Yoshimura, Y. , & Otani, H. (2007). Fetal jaw movement affects development of articular disk in the temporomandibular joint. Congenital anomalies, 47, 53–57. 17504387 10.1111/j.1741-4520.2007.00143.x

[ede12426-bib-0046] Harpman, J. A. , & Woollard, H. H. (1938). The tendon of the lateral pterygoid muscle. Journal of Anatomy, 73, 112–115. 17104737 PMC1252532

[ede12426-bib-0047] Hinton, R. J. (2014). Genes that regulate morphogenesis and growth of the temporomandibular joint: A review. Developmental Dynamics, 243, 864–874. 24668501 10.1002/dvdy.24130

[ede12426-bib-0048] Hinton, R. J. , Jing, J. , & Feng, J. Q. (2015). Genetic influences on temporomandibular joint development and growth. Craniofacial Development, 115, 85–109. 10.1016/bs.ctdb.2015.07.008 26589922

[ede12426-bib-0116] Hinton, R. J. , Jing, Y. , Jing, J. , & Feng, J. Q. (2016). Roles of chondrocytes in endochondral bone formation and fracture repair. Journal of Dental Research, 96(1), 23–30. 27664203 10.1177/0022034516668321 PMC5347428

[ede12426-bib-0049] Hirouchi, H. , Kitamura, K. , Yamamoto, M. , Odaka, K. , Matsunaga, S. , Sakiyama, K. , & Abe, S. (2017). Developmental characteristics of secondary cartilage in the mandibular condyle and sphenoid bone in mice. Archives of Oral Biology, 89, 84–92. 29494810 10.1016/j.archoralbio.2017.12.027

[ede12426-bib-0050] Howenstine, A. O. , Sadier, A. , Anthwal, N. , Lau, C. L. , & Sears, K. E. (2021). Non‐model systems in mammalian forelimb evo‐devo. Current Opinion in Genetics & Development, 69, 65–71. 33684847 10.1016/j.gde.2021.01.012 PMC8364859

[ede12426-bib-0051] Jäger, K. R. K. , Cifelli, R. L. , & Martin, T. (2020). Molar occlusion and jaw roll in early crown mammals. Scientific Reports, 10, 22378. 33361774 10.1038/s41598-020-79159-4 PMC7759581

[ede12426-bib-0052] Jing, Y. , Zhou, X. , Han, X. , Jing, J. , von der Mark, K. , Wang, J. , de Crombrugghe, B. , Hinton, R. J. , & Feng, J. Q. (2015). Chondrocytes directly transform into bone cells in mandibular condyle growth. Journal of Dental Research, 94(12), 1668–1675. 26341973 10.1177/0022034515598135 PMC4681473

[ede12426-bib-0053] Kantomaa, T. , & Hall, B. K. (1988). Mechanism of adaptation in the mandibular condyle of the mouse. Cells Tissues Organs, 132(2), 114–119. 10.1159/000146561 3414355

[ede12426-bib-0054] Kemp, T. S. (1979). The primitive cynodont procynosuchus: Functional anatomy of the skull and relationships. Philosophical Transactions of the Royal Society B: Biological Sciences, 285, 73–122.

[ede12426-bib-0055] Kemp, T. S. (2005). The origin and evolution of mammals. Oxford University Press.

[ede12426-bib-0056] Kermack, K. A. , Mussett, F. , & Rigney, H. W. (1973). The lower jaw of morganucodon. Zoological Journal of the Linnean Society, 53(2), 87–175.

[ede12426-bib-0057] Kult, S. , Olender, T. , Osterwalder, M. , Markman, S. , Leshkowitz, D. , Krief, S. , Blecher‐Gonen, R. , Ben‐Moshe, S. , Farack, L. , Keren‐Shaul, H. , Salame, T. M. , Capellini, T. D. , Itzkovitz, S. , Amit, I. , Visel, A. , & Zelzer, E. (2021). Bi‐fated tendon‐to‐bone attachment cells are regulated by shared enhancers and KLF transcription factors. eLife, 10, e55361. 33448926 10.7554/eLife.55361 PMC7810463

[ede12426-bib-0058] Lautenschlager, S. , Gill, P. , Luo, Z.‐X. , Fagan, M. J. , & Rayfield, E. J. (2017). Morphological evolution of the mammalian jaw adductor complex. Biological Reviews, 92, 1910–1940. 27878942 10.1111/brv.12314 PMC6849872

[ede12426-bib-0059] Li, N. , Hu, P. , Wang, Y. , Chen, X. , Wang, S. , Shi, Y. , Huang, Z. , Lin, C. , Zhang, Y. , Cong, W. , Xiao, J. , & Liu, C. (2021). Tissue interactions are indispensable for cavity formation and disc separation in the temporomandibular joint. Connective Tissue Research, 62, 351–358. 31875727 10.1080/03008207.2019.1709452

[ede12426-bib-0060] Love, A. (2006). Evolutionary morphology and evo‐devo: Hierarchy and novelty. Theory in Biosciences, 124, 317–333. 17046363 10.1016/j.thbio.2005.11.006

[ede12426-bib-0061] Luo, Z.‐X. , Schultz, J. A. , & Ekdale, E. G. (2016). Evolution of the vertebrate ear. Springer International Publishing.

[ede12426-bib-0062] Luo, Z.‐X. (2011). Developmental patterns in mesozoic evolution of mammal ears. Annual Review of Ecology, Evolution, and Systematics, 42, 355–380.

[ede12426-bib-0063] Ma, C. , Jing, Y. , Li, H. , Wang, K. , Wang, Z. , Xu, C. , Sun, X. , Kaji, D. , Han, X. , Huang, A. , & Feng, J. Q. (2021). ScxLin cells directly form a subset of chondrocytes in temporomandibular joint that are sharply increased in Dmp1‐null mice. Bone, 142, 115687. 33059101 10.1016/j.bone.2020.115687 PMC7749445

[ede12426-bib-0064] Mérida‐Velasco, J. R. , Rodríguez‐Vázquez, J. F. , de la Cuadra Blanco, C. , Campos López, R. , Sánchez, M. , & Mérida Velasco, J. A. (2009). Development of the mandibular condylar cartilage in human specimens of 10–15 weeks' gestation. Journal of Anatomy, 214, 56–64. 19166473 10.1111/j.1469-7580.2008.01009.x PMC2667917

[ede12426-bib-0065] Mérida‐Velasco, J. R. , Rodríguez‐Vázquez, J. F. , Mérida‐Velasco, J. A. , Sanchez‐Montesinos, I. , Espín‐Ferra, J. , & Jiménez‐Collado, J. (1999). Development of the human temporomandibular joint. The Anatomical Record, 255, 20–33. 10321990 10.1002/(SICI)1097-0185(19990501)255:1<20::AID-AR4>3.0.CO;2-N

[ede12426-bib-0066] Michailovici, I. , Eigler, T. , & Tzahor, E. (2015). Craniofacial muscle development. Elsevier Inc. 10.1016/bs.ctdb.2015.07.022 26589919

[ede12426-bib-0067] Moczek, A. P. (2008). On the origins of novelty in development and evolution. BioEssays, 30, 432–447. 18404691 10.1002/bies.20754

[ede12426-bib-0068] Muller, G. B. , & Wagner, G. P. (1991). Novelty in evolution: Restructuring the concept. Annual Review of Ecology and Systematics, 22, 229–256.

[ede12426-bib-0069] Naples, V. L. (1999). Morphology, evolution and function of feeding in the giant anteater (*Myrmecophaga tridactyla*). Journal of Zoology, 249, 19–41.

[ede12426-bib-0070] Ögütcen‐Toller, M. , & Juniper, R. P. (1993). The embryologic development of the human lateral pterygoid muscle and its relationships with the temporomandibular joint disc and Meckel's cartilage. Journal of Oral and Maxillofacial Surgery, 51, 772–778. 8509918 10.1016/s0278-2391(10)80420-3

[ede12426-bib-0071] Öğütcen‐Toller, M. , & Keskin, M. (2000). Computerized 3‐dimensional study of the embryologic development of the human masticatory muscles and temporomandibular joint. Journal of Oral and Maxillofacial Surgery, 58, 1381–1386. 11117686 10.1053/joms.2000.18270

[ede12426-bib-0072] Oka, K. , Oka, S. , Sasaki, T. , Ito, Y. , Bringas, P. , Nonaka, K. , & Chai, Y. (2007). The role of TGF‐β signaling in regulating chondrogenesis and osteogenesis during mandibular development. Developmental Biology, 303, 391–404. 17204263 10.1016/j.ydbio.2006.11.025 PMC2074881

[ede12426-bib-0073] Peters, H. , Neubüser, A. , Kratochwil, K. , & Balling, R. (1998). Pax9‐deficient mice lack pharyngeal pouch derivatives and teeth and exhibit craniofacial and limb abnormalities. Genes & Development, 12, 2735–2747. 9732271 10.1101/gad.12.17.2735 PMC317134

[ede12426-bib-0074] Pinto, O. F. (1962). A new structure related to the temporomandibular joint and middle ear. The Journal of Prosthetic Dentistry, 12, 95–103.

[ede12426-bib-0075] Purcell, P. , Jheon, A. , Vivero, M. P. , Rahimi, H. , Joo, A. , & Klein, O. D. (2012). Spry1 and spry2 are essential for development of the temporomandibular joint. Journal of Dental Research, 91, 387–393. 22328578 10.1177/0022034512438401 PMC3310757

[ede12426-bib-0076] Purcell, P. , Joo, B. W. , Hu, J. K. , Tran, P. v , Calicchio, M. L. , O'Connell, D. J. , Maas, R. L. , & Tabin, C. J. (2009). Temporomandibular joint formation requires two distinct Hedgehog‐dependent steps. Proceedings of the National Academy of Sciences of the United States of America, 106, 18297–18302. 19815519 10.1073/pnas.0908836106 PMC2775291

[ede12426-bib-0077] Ramírez‐Chaves, H. E. , Wroe, S. W. , Selwood, L. , Hinds, L. A. , Leigh, C. , Koyabu, D. , Kardjilov, N. , & Weisbecker, V. (2016). Mammalian development does not recapitulate suspected key transformations in the evolutionary detachment of the mammalian middle ear. Proceedings of the Royal Society B: Biological Sciences, 283(1822), 20152606. 10.1098/rspb.2015.2606 PMC4721106 26763693

[ede12426-bib-0078] Reilly, S. M. , McBrayer, L. D. , & White, T. D. (2001). Prey processing in amniotes: Biomechanical and behavioral patterns of food reduction. Comparative Biochemistry and Physiology Part A: Molecular & Integrative Physiology, 128, 397–415. 10.1016/s1095-6433(00)00326-3 11246036

[ede12426-bib-0079] Roberts, R. R. , Bobzin, L. , Teng, C. S. , Pal, D. , Tuzon, C. T. , Schweitzer, R. , & Merrill, A. E. (2019). FGF signaling patterns cell fate at the interface between tendon and bone. Development, 146, dev170241. 31320326 10.1242/dev.170241 PMC6703712

[ede12426-bib-0080] Roberts, W. E. , & Stocum, D. L. (2018). Part II: Temporomandibular joint (TMJ)—Regeneration, degeneration, and adaptation. Current Osteoporosis Reports, 16, 369–379. 29943316 10.1007/s11914-018-0462-8

[ede12426-bib-0081] Rodríguez‐Vázquez, J. F. , Mérida‐Velasco, J. R. , Mérida‐Velasco, J. A. , & Jiménez‐Collado, J. (1998). Anatomical considerations on the discomalleolar ligament. Journal of Anatomy, 192, 617–621. 9723988 10.1046/j.1469-7580.1998.19240617.x PMC1467815

[ede12426-bib-0082] Romer, A. S. (1969). Cynodont reptile with incipient mammalian jaw articulation. Science, 166(3907), 881–882. 5345204 10.1126/science.166.3907.881

[ede12426-bib-0083] Rot‐Nikcevic, I. , Downing, K. J. , Hall, B. K. , & Kablar, B. (2007). Development of the mouse mandibles and clavicles in the absence of skeletal myogenesis. Histology and Histopathology, 22, 51–60. 17128411 10.14670/HH-22.51

[ede12426-bib-0084] Ruscitto, A. , Morel, M. M. , Shawber, C. J. , Reeve, G. , Lecholop, M. K. , Bonthius, D. , Yao, H. , & Embree, M. C. (2020). Evidence of vasculature and chondrocyte to osteoblast transdifferentiation in craniofacial synovial joints: Implications for osteoarthritis diagnosis and therapy. The FASEB Journal, 34, 4445–4461. 32030828 10.1096/fj.201902287R PMC7380713

[ede12426-bib-0085] Schneider, R. A. (2018). Neural crest and the origin of species‐specific pattern. Genesis, 56(6–7), e23219. 30134069 10.1002/dvg.23219 PMC6108449

[ede12426-bib-0086] Schneider, R. A. , & Helms, J. A. (2003). The cellular and molecular origins of beak morphology. Science, 299(5606), 565–568. 12543976 10.1126/science.1077827

[ede12426-bib-0087] Schwartz, A. G. , Long, F. , & Thomopoulos, S. (2015). Enthesis fibrocartilage cells originate from a population of Hedgehog‐responsive cells modulated by the loading environment. Development, 142, 196–206. 25516975 10.1242/dev.112714 PMC4299149

[ede12426-bib-0088] Schweitzer, R. , Chyung, J. H. , Murtaugh, L. C. , Brent, A. E. , Rosen, V. , Olson, E. N. , Lassar, A. , & Tabin, C. J. (2001). Analysis of the tendon cell fate using Scleraxis, a specific marker for tendons and ligaments. Development, 128, 3855–3866. 11585810 10.1242/dev.128.19.3855

[ede12426-bib-0089] Shibata, S. , Sato, R. , Murakami, G. , Fukuoka, H. , & Francisco Rodríguez‐Vázquez, J. (2013). Origin of mandibular condylar cartilage in mice, rats, and humans: Periosteum or separate blastema? Journal of Oral Biosciences, 55, 208–216.

[ede12426-bib-0090] Shibata, S. , Suda, N. , Yoda, S. , Fukuoka, H. , Ohyama, K. , Yamashita, Y. , & Komori, T. (2004). Runx2‐deficient mice lack mandibular condylar cartilage and have deformed Meckel's cartilage. Anatomy and Embryology, 208, 273–280. 15156401 10.1007/s00429-004-0393-2

[ede12426-bib-0091] Shibata, S. , & Yokohama‐Tamaki, T. (2008). An in situ hybridization study of Runx2, Osterix, and Sox9 in the anlagen of mouse mandibular condylar cartilage in the early stages of embryogenesis. Journal of Anatomy, 213(3), 274–283. 18624832 10.1111/j.1469-7580.2008.00934.x PMC2732041

[ede12426-bib-0092] Shibukawa, Y. , Young, B. , Wu, C. , Yamada, S. , Long, F. , Pacifici, M. , & Koyama, E. (2007). Temporomandibular joint formation and condyle growth require Indian Hedgehog signaling. Developmental Dynamics, 236(2), 426–434. 17191253 10.1002/dvdy.21036

[ede12426-bib-0093] Shubin, N. , Tabin, C. , & Carroll, S. (2009). Deep homology and the origins of evolutionary novelty. Nature, 457, 818–823. 19212399 10.1038/nature07891

[ede12426-bib-0094] Sidor, C. A. (2003). Evolutionary trends and the origin of the mammalian lower jaw. Paleobiology, 29, 605–640.

[ede12426-bib-0095] Solem, R. C. , Eames, B. F. , Tokita, M. , & Schneider, R. A. (2011). Mesenchymal and mechanical mechanisms of secondary cartilage induction. Developmental Biology, 356, 28–39. 21600197 10.1016/j.ydbio.2011.05.003 PMC3130809

[ede12426-bib-0096] Stocum, D. L. , & Roberts, W. E. (2018). Part I: Development and physiology of the temporomandibular joint. Current osteoporosis reports, 16, 360–368. 29948821 10.1007/s11914-018-0447-7

[ede12426-bib-0097] Sugimoto, Y. , Takimoto, A. , Akiyama, H. , Kist, R. , Scherer, G. , Nakamura, T. , Hiraki, Y. , & Shukunami, C. (2013). Scx+/Sox9+ progenitors contribute to the establishment of the junction between cartilage and tendon/ligament. Development, 140, 2280–2288. 23615282 10.1242/dev.096354

[ede12426-bib-0098] Svandova, E. , Anthwal, N. , Tucker, A. S. , & Matalova, E. (2020). Diverse fate of an enigmatic structure: 200 years of Meckel's cartilage. Frontiers in Cell and Developmental Biology, 8, 821. 32984323 10.3389/fcell.2020.00821 PMC7484903

[ede12426-bib-0099] Tokita, M. , & Schneider, R. A. (2009). Developmental origins of species‐specific muscle pattern. Developmental Biology, 331(2), 311–325. 19450573 10.1016/j.ydbio.2009.05.548 PMC2726847

[ede12426-bib-0100] Tucker, A. S. , & Lumsden, A. (2004). Neural crest cells provide species‐specific patterning information in the developing branchial skeleton. Evolution and Development, 6(1), 32–40. 15108816 10.1111/j.1525-142x.2004.04004.x

[ede12426-bib-0101] Tucker, A. S. , Watson, R. P. , Lettice, L. A. , Yamada, G. , & Hill, R. E. (2004). Bapx1 regulates patterning in the middle ear: Altered regulatory role in the transition from the proximal jaw during vertebrate evolution. Development, 131, 1235–1245. 14973294 10.1242/dev.01017

[ede12426-bib-0102] Urban, D. J. , Anthwal, N. , Luo, Z.‐X. , Maier, J. A. , Sadier, A. , Tucker, A. S. , & Sears, K. E. (2017). A new developmental mechanism for the separation of the mammalian middle ear ossicles from the jaw. Proceedings of the Royal Society B: Biological Sciences, 284, 20162416. 10.1098/rspb.2016.2416 PMC5310609 28179517

[ede12426-bib-0103] Vinkka, H. (1982). Secondary cartilages in the facial skeleton of the rat. Proceedings of the Finnish Dental Society. Suomen Hammaslaakariseuran Toimituksia, 78(Suppl 7), 1–137. 7184017

[ede12426-bib-0104] Vinkka‐Puhakka, H. , & Thesleff, I. (1993). Initiation of secondary cartilage in the mandible of the Syrian hamster in the absence of muscle function. Archives of Oral Biology, 38, 49–54. 8442720 10.1016/0003-9969(93)90154-e

[ede12426-bib-0105] Wang, Y. , Liu, C. , Rohr, J. , Liu, H. , He, F. , Yu, J. , Sun, C. , Li, L. , Gu, S. , & Chen, Y. (2011). Tissue interaction is required for glenoid fossa development during temporomandibular joint formation. Developmental Dynamics, 240, 2466–2473. 21953591 10.1002/dvdy.22748 PMC3197963

[ede12426-bib-0106] Watson, D. M. S. (1916). The monotreme skull: A contribution to mammalian morphogenesis. Philosophical Transactions of the Royal Society of London. Series B, Containing Papers of a Biological Character, 207(1916), 311–374.

[ede12426-bib-0107] Woronowicz, K. C. , Gline, S. E. , Herfat, S. T. , Fields, A. J. , & Schneider, R. A. (2018). FGF and TGFβ signaling link form and function during jaw development and evolution. Developmental Biology, 444, S219–S236. 29753626 10.1016/j.ydbio.2018.05.002 PMC6239991

[ede12426-bib-0108] Xiang, L. , Wang, X. , Li, Y. , Liu, H. W. , Zhang, X. , Mu, X. , Liu, C. , & Hu, M. (2022). Development of the temporomandibular joint in miniature pig embryos. Journal of Morphology, 283, 134–143. 34800049 10.1002/jmor.21432

[ede12426-bib-0109] Zeller, U. (1993). Ontogenetic evidence for cranial homologies in monotremes and therians, with special reference to *Ornithorhynchus* . In F. S. Szalay , M. J. Novacek , & M. C. McKenna (Eds.), Mammal phylogeny (pp. 95–107). Springer.

[ede12426-bib-0110] Zhang, S. , Yap, A. U. J. , & Toh, W. S. (2015). Stem cells for temporomandibular joint repair and regeneration. Stem Cell Reviews and Reports, 11, 728–742. 26123357 10.1007/s12015-015-9604-x

[ede12426-bib-0111] Zhou, C.‐F. , Wu, S. , Martin, T. , & Luo, Z.‐X. (2013). A Jurassic mammaliaform and the earliest mammalian evolutionary adaptations. Nature, 500(7461), 163–167. 23925238 10.1038/nature12429

[ede12426-bib-0112] Ziermann, J. M. , Diogo, R. , & Noden, D. M. (2018). Neural crest and the patterning of vertebrate craniofacial muscles. Genesis, 56, e23097. 29659153 10.1002/dvg.23097

